# Mediating Role of Rumination Between Anger and Anxious-Depressive Symptomatology in Family Members of People with Gambling Disorder

**DOI:** 10.1007/s10899-022-10178-8

**Published:** 2022-12-26

**Authors:** A. Estévez, P. Jauregui, J. Momeñe, L. Macía, N. Etxaburu

**Affiliations:** Psychology Department, University of Deusto, Apartado 1, 48080 Bilbao, Spain

**Keywords:** Gambling, Family, Rumination, Anger, Anxiety, Depression

## Abstract

Gambling disorder is characterized by a behavioural pattern of dysfunctional gambling that persists despite its negative implications in different areas of people’s daily life. One of the most negatively affected areas is the one related to family members. This study aimed, firstly, to study the differences between family members of people with gambling disorder and a general population sample in anger (state, trait, expression-out, expression-in. control-out and control-in), rumination (brooding, reflection and total), and anxiety and depression. The second aim was to analyse the correlation between these variables in the family members of people with gambling disorder, and thirdly, to analyse the mediating role of rumination between anger, anxiety and depression. This study consisted of 170 people, of whom 87 were family members of people with a gambling disorder, and 83 were from the general population. Instruments measuring anger, anxiety, depression, and ruminative responses were administered. Results showed that family members had significantly higher scores in anger (state), depression, anxiety, rumination (total and brooding). Also, results showed that anger correlated positively and significantly with rumination, depression and anxiety, which also correlated positively and significantly with each other. Third, rumination mediated the relationship between the following variables: anger (state) and depression; anger (trait) and anxiety and depression; anger (external expression) and anxiety and depression. A complete mediating effect was found in the latter case and a partial mediating effect in the first two cases. In conclusion, it is found that having a family member with a gambling disorder may increase levels of anger, anxiety, depression and rumination. Furthermore, it is shown that working on rumination may reduce depression and anxiety in family members of gamblers.

## Introduction

Gambling disorder (GD) is characterized by a behavioural pattern of dysfunctional gambling that persists despite its negative implications in different areas of people’s daily life (Ferrara et al., 2018). In the last few years, problem gambling behaviours have increased exponentially due to the current opportunities, their accessibility and availability. Moreover, gambling has changed from being perceived as having negative connotations to being a socially accepted pastime (Derevensky et al., 2019).

One of the most negatively affected areas is the one related to family members (Dighton et al., 2018). Partners of people suffering from gambling disorder (GD) report increased family dysfunction (Black et al., 2012), as well as relational conflicts and tensions (Jeffrey et al., 2019) that frequently lead to the couple’s breakup or divorce (Atherton & Beynon, 2019). They also continuously cope with the damage of gambling-related problems on family life (Petra, 2020). In this sense, ambivalent feelings are also common, some gamblers' partners want to give their family a chance, while others are concerned about the well-being of their children and decide to divorce the gambler (Järvinen-Tassopoulos, 2020). Some studies have investigated how family members of people with GD deal with the impact or the negative effects of this disorder on them (Chan et al., 2016; Dowling et al., 2014; Estevez et al., 2020) and the potential benefits of family involvement in the treatment both for the person with GD and the family have been noted (Kourgiantakis et al., 2018). After all, family members are also indirectly involved in the consequences that this problem generates in several areas of daily functioning, such as the psychological area (Melero et al., 2019). They may also have an effect on the development of this addiction (Mazar et al., 2018). This is due to the concept of circularity, where all behaviours emitted by one member of the family will influence the behaviours of the rest of the family members and vice versa (Páez-Cala, 2019).

Regarding the psychological area, the pathological gambler´s family members, in general, go through three different stages: negation, stress, and exhaustion (Custer & Milt, 1985). They also tend to show several emotional problems derived from chronic gambling-related stress, financial losses, mistrust, continuous family conflicts and lack of communication, with particular emphasis on anxiety and depression (Chan et al., 2016) and anger, which can sometimes lead to violence against the gambler. Qualitative studies show that anger may be triggered as a result of gambling-related conflicts in pathological gambler’s family members (Suomi et al., 2013). However, it has also been noted that some people gamble as a way of coping with the family violence they have suffered (Dowling, 2014). Anger is known to be a destructive emotion with significant social and public health relevance. It arises in situations that are interpreted as being provocative and cause a rapid onset of autonomic arousal (Painuly et al., 2005). It has been shown that anger can have a great influence on the performance of maladaptive behaviours (de Bles et al., 2019).

One of the mechanisms that may help to understand the impact and incidence both of anger, depression and anxiety is rumination. Rumination can sometimes precede psychopathology and consists of a repetitive, intrusive and unwanted thought pattern, in response to the distress involved in the origin and consequences of an unpleasant event or emotional experience (du Pont et al., 2019; García et al., 2017). Thus, rumination is a maladaptive emotion-regulation mechanism (Schäfer et al., 2017), characterised by an excessive focus on the negative thoughts and feelings associated with a relevant or negative event (Garnefski & Kraaij, 2007). Numerous studies have examined the relationship between rumination and anger, finding that rumination tends to persist for long periods of time, which, when coexisting with high levels of anger, could lead to increased frustration (Takebe et al., 2016). Likewise, the constant rumination on emotions such as anger predicts the onset and maintenance of anxiety and impulsive or maladaptive behaviour (Law et al., 2021), such as gambling. Links between rumination, anger and depression have also been found (Balsamo, 2010), as rumination on emotions such as anger over the years predicts the development of higher levels of depression and anxiety (Izadpanah et al., 2017). Previous studies have reported a close relationship between anger rumination and depressive rumination, both predicting internalising and externalising psychopathology across the lifespan (du Pont et al., 2018). In this line, another study has found an association between anger and depression through anger rumination (Besharat et al., 2013). Furthermore, depressive rumination is a vulnerability factor in the aetiology, maintenance and recurrence of depression (Ricarte et al., 2018; Sirota et al., 2019). Previous studies, in turn, have found that rumination on anger is related to the presence of problems such as major depressive disorder (Besharat et al., 2013). This could be because the dysfunctional nature of rumination predicts maladaptative problem-solving, as well as the maintenance and enhancement of negative stressors and increased major depression (De Rosa & Keegan, 2018). Therefore, rumination is a significant process in major depression (Kovács et al., 2020). Consequently, one might expect anger to appear first, then rumination on the anger, which then triggers anxious and depressive symptomatology.

As it has been pointed out, different studies have shown that anger, anxiety and depression may be present in family members of people with GD but a few studies have analysed their relationship and the underlying explanatory mechanisms. Therefore, this study aimed, firstly, to study the differences between family members of people with GD and a general population sample in anger, rumination, anxiety and depression. A second aim was to analyse the correlation between these variables in the clinical sample, and thirdly, to analyse the mediating role of rumination between anger, anxiety and depression.

## Methods

### Participants

This sample consisted of 170 people, of whom 87 were family members of people with GD, and 83 were from the general population. An inclusion criterion for the group of family members was to have had a family member with a gambling problem and to attend a treatment center for GD, whereas this was an exclusion criterion for the general population sample. In both groups, being of legal age (over 18 years old) was also a requirement to be able to participate in the study (Table 1).

**Table 1:** Description of the two sample groups

	Family members of people with gambling disorder	Not family members of people with gambling addiction
Sample size	87 participants	83 participants
Age	20–75 years old (*M* = 52,18, *SD* = 13,15)	18–70 years old (*M* = 42,28, *SD* = 17,36)
Sex	22 men (25,3%), 65 women (74,7%)	31 men (37,3%), 51 women (61,4%) and one person did not specify sex (1,2%)
Marital status	56 (64.4%) are married, 16 (18.4%) are single, 8 (9.2%) are separated or divorced, 5 (5.7%) are widowed and 2 (2.3%) are in a civil partnership	29 (34.9%) are married, 41 (49.4%) are single, 6 (7.2%) are separated or divorced, 3 (3.6%) are widowed and 4 (4.8%) are in a civil partnership
Leve lof education	24 (27.6%) have primary education, 17 (19.5%) have secondary education, 20 (23%) have vocational training, 25 (28.17%) have university education and 1 (1.1%) did not answer	6 (7.2%) have primary education, 15 (18.1%) have secondary education, 15 (18.1%) have vocational training and 47 (56.6%) have university studies
Employement status	47 (54%) are currently working, 7 (8.1%) are unemployed, 4 (4.7%) are studying, 24 (27.9%) are retired and 4 (4.7%) are on sick leave	49 (59.8%) are currently working, 3 (3.6%) are unemployed, 19 (22.9%) are studying, 9 (10.8%) are retired, 2 (2.4%) are working and studying and 1 (1.2%) did not answer
Kinship to the person with gambling disorder	10 (11.8%) participants are the gambler's wife or husband, 9 (10.6%) are the gambler's siblings, 4 (4.6%) are the gambler's sons or daughters, 9 (10.3%) are the gambler's partner, 48 (56, 5%) are fathers or mothers of the gambler, 5 (5.9%) have another type of relationship with the gambler, being grandfather of the gambler 2 (2.3%), brother-in-law of the gambler 2 (2.3%) or uncle of the gambler 1 (1.1%)	

The sample of gamblers' family members was drawn from a treatment center for problem gamblers. This center is associated with the Spanish Federation of Rehabilitated Gamblers and there is psychological care for gambling problems and there are also groups of relatives who receive treatment. Family members were asked to take part in the study. For the general population sample, family members were asked to look for people in their environment who did not have family members and who had similar characteristics.

### Instruments

**State-Trait Anger Expression Inventory-2 (STAXI-2**; Spielberger, 1999; adapted by Miguel-Tobal et al., 2009). The STAXI-2 State Anger scale assesses the intensity of anger as an emotional state at a particular time. It has 3 subscales: Feeling angry, Feeling like expressing anger verbally, Feeling like expressing anger physically. The Trait Anger scale measures how often angry feelings are experienced over time. It has 2 subscales: Angry Temperament and Angry Reaction. The Anger Expression and Anger Control scales assess four relatively independent anger-related traits: (a) Anger Expression-Out: expression of anger toward other persons or objects in the environment; (b) Anger Expression-In: holding in or suppressing angry feelings; (c) Anger Control-Out: controlling angry feelings by preventing the expression of anger toward other persons or objects in the environment and (d) Anger Control-In: controlling suppressed angry feelings by calming down or cooling off Anger Expression Index: Overall measure of expression/control, it is based on The Anger Expression and Anger Control Scales.

Individuals rate themselves on 4-point scales (1. Almost never; 2. Sometimes; 3. Often; 4. Almost always) that assess both the intensity of their anger at a particular time and the frequency that anger is experienced, expressed, and controlled. It provides an overall index of the frequency with which anger is expressed, regardless of the direction of expression (external, internal). In the current study, Cronbach's alpha was 0.88.

**Symptom Checklist (SCL-90-R;** Derogatis, 2002). This inventory was developed to assess patterns of symptoms present in individuals and can be used both in community samples and clinical diagnostic tasks. It is composed of 90 items, each of which describes a specific psychopathological or psychosomatic disturbance: Somatisations, Obsessions and Compulsions, Interpersonal Sensitivity, Depression, Anxiety, Hostility, Phobic Anxiety, Paranoid Ideation, and Psychoticism. These nine dimensions are grouped into three global indices of psychological distress. The first is the Global Severity Index (GSI) which refers to the average intensity of all 90 items. The second is the Total Positive Symptoms, which refers to the average number of symptoms experienced by each person. And the third is the Positive Symptom Distress Index (PSDI), which refers to the average intensity of positive symptoms. The intensity of distress caused by each symptom should be rated by the respondent from 0 (*total absence of symptom-related distress*) to 4 (*maximum distress*).

The internal consistency coefficient for each of the SCL-90 scales was high (0.90) and it presented good test–retest reliability over a two-week period (Derogatis, 1983, 2002). In this study, only the anxiety and depression scales were considered since these are disorders that different studies have found in family members of people with gambling disorder (Estevez et al., 2020; Shaw et al, 2007). Cronbach's alpha was 0.92 for the anxiety subscale and 0.91 for the depression subscale.

**Ruminative Responses Scale** (**RRS**; Nolen-Hoeksema, 1991; Spanish adaptation by Hervas, 2008). This scale assesses the presence of the ruminative response style, a response pattern that consists of an excessive focus on the causes and consequences of depressive symptoms. Ruminative style has been associated with an increased likelihood of developing depressive and anxious symptomatology (Treynor et al., 2003). It is composed by 22 items divided into two factors, Brooding and Reflection, with Brooding being highly maladaptive, and Reflection being adaptive (Teasdale & Green, 2004). The response options correspond to a 5-point Likert scale from "*Strongly disagree*" to "*Strongly agree*". Therefore, scores can range from 22 to 110 points.

Regarding the psychometric properties of the full version of the Ruminant Responses Scale, several studies have shown that it has good internal consistency as well as adequate test–retest reliability (Nolen-Hoeksema et al., 1994, 1999). In the current study, Cronbach's alpha was 0.94.

### Procedure

The clinical sample was collected through gambling disorder treatment center, while the general population sample was collected through convenience sampling. The clinical sample completed the questionnaires presentially, while the general population sample completed the questionnaires online. In all cases, the researchers invited participants to take part in the study and explained its purpose. Confidentiality, anonymity were guaranteed, and informed consent to participate was required. The contact details of the study's reference researchers were also provided. The total duration of sample collection was one year. This study has the approval of the Ethics Committee of the University of Deusto ETK-26/17-18.

### Data analysis

First, mean differences between the sample of family members and non-family members in anger, rumination, anxiety, and depression were analyzed using Student's *t*-test. Next, effect size was measured for determining the magnitude of the difference among the variables in both samples, independently from the sample size, using Cohen's *d*, whose parameters state that a value of 0.20 corresponds to a small effect size, around 0.50 is medium, and above 0.80 is large (Cohen, 1992). No power analysis was conducted to previously estimate the sample size, since it was a convenience sampling. Secondly, bivariate relationships between anger, rumination, anxiety, and depression in the sample of family members of gamblers were analyzed through Pearson's correlation analyses (*r*).

Finally, the mediational role of rumination (M) between anger (independent variable X) and anxiety and depression (dependent variable Y) was analyzed through multiple mediation analyses, controlling for the effect of age, sex, and kinship type. A model with each one of the anger components (state, trait, external control, internal control, external expression, and internal expression) was tested twice, once with depression and once with anxiety. Analyses were performed using the INDIRECT macro of Preacher and Hayes (2008), which is an adequate way to measure multiple mediation models with small and moderate samples. First, the significance of the effect of the independent variable on the mediator variables (a-path) and the effect of mediator variables on the dependent variable (b-path) were tested. Then, the total effect of the independent variable on the dependent variable along with the mediator variables (c-path) was measured, and the direct effect of the independent variable on the dependent variable was measured while controlling for the effect of mediator variables (c’-path). A full mediation effect is found when the c-path is significant but the c’-path is not, whereas a partial mediation effect is found when both the c and c’-path are significant.

## Results

First, mean differences were analysed among family members and non-family members through the *t*-test (Table 2). Results showed that family members had significantly higher scores in anger (State), depression, anxiety, and rumination (total and brooding). Among the statistically significant scales, a large effect size was found in rumination (brooding), and a medium one in anger (State), depression, anxiety, and rumination (total).

Second, bivariate relationships were tested in the sample of family members through Pearson’s *r* (Table 3)*.* Results showed that anger correlated positively and significantly with rumination, depression, and anxiety. More specifically, anger (State) and anger (In) correlated with all the rumination, anxiety, and depression scales. The same results were found for anger (Trait) and anger (Out expression) except for rumination (reflection), which did not significantly correlate with these scales. Anger Control-In and Anger Control-Out did not correlate with any other scale, except for internal control and rumination (brooding). In the case of rumination, anxiety, and depression, all the scales correlated significantly with each other.

**Table 2:** Comparison between family members and non-family members in anger, rumination, anxiety, and depression

Variables	Family members		Non-family members		*t*(*df*)	*d*
	*M*	*SD*	*M*	*SD*		
1. Anger - state	21.04	8.77	18.70	8.22	1.71 (1152)*	.28
2. Anger - trait	18.84	5.61	17.99	5.61	0.96 (1158)	.15
3. Anger expression-out	9.92	2.45	10.05	2.71	− 0.31 (1158)	− .05
4. Anger expression in	12.03	3.64	11.58	3.46	0.79 (1153)	.13
5. Anger control-out	16.42	4.43	17.75	4.06	− 1.94 (1152)	− .31
6. Anger control in	14.67	4.73	14.73	3.78	− 0.09 (1149.087)	− .01
7. Depression	17.96	10.94	12.96	11.44	2.79 (1154)*	.45
8. Anxiety	12.65	10.54	8.30	8.94	2.73 (1143.133)*	.45
9. Rumination - reflection	9.48	3.49	8.88	3.09	1.10 (1145)	.18
10. Rumination - boording	10.42	3.41	8.27	3.03	4.12 (1152)*	.67
11. Rumination - total	42.88	12.76	38.54	12.80	2.014 (1139)*	.34

*p < .05

**Table 3:** Correlations between anger, rumination, anxiety, and depression in family members of gamblers

Variables	Rumination - reflection	Rumination – boording	Rumination – Total	Depression	Anxiety
1. State anger	.25^*^	.62^*^	.55^*^	.60^*^	.63^*^
2. Trait anger	.13	.35^*^	.34^*^	.34^*^	.46^*^
3. Anger expression-out	.19	.45^*^	.42^*^	.28^*^	.38^*^
4. Anger expression in	.42^*^	.44^*^	.46^*^	.34^*^	.21
5. Anger control-out	.14	− .19	− .17	− .06	− .15
6. Anger control in	.18	− .24^*^	− .18	− .11	− .16

*p = < .05

**Fig. 1:**
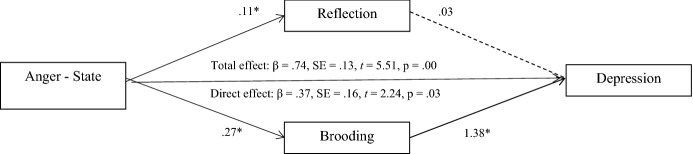
Mediating role of rumination among anger (state) and depression

**Fig. 2:**
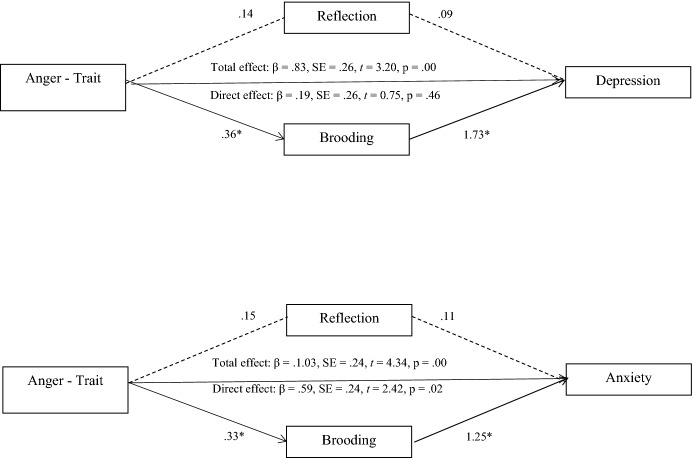
Mediating role of rumination among anger (trait) and anxiety and depression

**Fig. 3:**
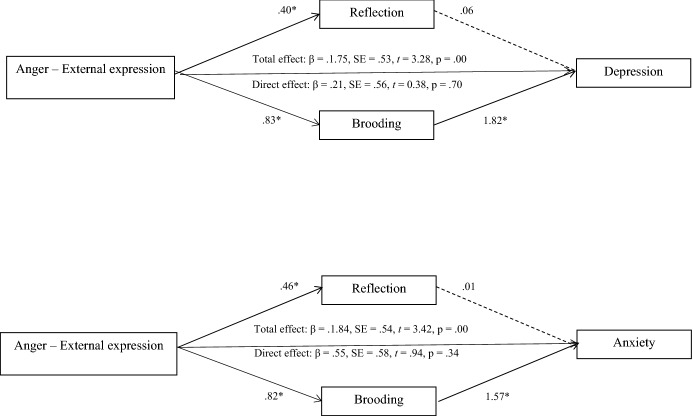
Mediating role of rumination among anger (External expression) and anxiety and depression

Third, the mediating role of rumination between anger, anxiety, and depression, was analyzed in the sample of family members through multiple mediation analyses (Preacher & Hayes, 2008). Results showed that rumination mediated the relationship between the following variables: anger (State) and depression; anger (Trait) and anxiety and depression; anger (Out), anxiety, and depression. A full mediating effect was found in the case of anger (Out) with anxiety and depression, and in the case of anger (Trait) with depression, whereas a partial mediating effect was found in anger (State) and depression, and in anger (Trait) and anxiety. In all the tested models, brooding was the variable that stood out as the significant mediator between anger, anxiety, and depression. Sex, age, and type of kinship were included as covariates to control for their effect, but no significant effect of these variables was found (Figs. 1, 2, 3).

## Discussion

This study first aimed to compare clinical samples of family members of people with pathological gambling with general population samples on anger, rumination, anxiety, and depression. First, it was found that family members of people with pathological gambling scored higher on anger (State), depression, and anxiety, and on rumination (total and brooding). The study of the emotional state of family members of people with gambling addiction is of great relevance due to the concept of circularity, that is, being social beings, all behaviors emitted by a family member will influence the behaviors of the rest of the family members and vice versa (Páez-Cala, 2019). Concerning this, Dowling (2014) mentions that family members of people with a gambling problem report high levels of anxiety, depression and anger and this could be due to being subjected to chronic stress for long periods of time due to financial losses, continuous conflicts, lack of communication, emotions surrounding the gambler, such as distrust and uncertainty. Studies such as Estevez et al. (2020) also find higher levels of anxiety and depression in family members of gamblers than in the general population. For their part, these results are in line with other studies that address the influence of other non-substance addictions, such as eating disorders, on the family members. Ochoa de Alda et al. (2006) found that family members of people with an eating disorder have higher scores in anxiety and depression compared to the control group. The results obtained in the present study also highlight that, in the case of anger, the variable in which significant differences were found was anger (State), suggesting that family members would show higher levels of anger at certain moments, but they would not show a greater tendency or predisposition to feel anger or more difficulties in expressing and managing anger than the general population. This result might suggest that circumstances related to the current gambling situation could be the trigger for the emotional difficulties reported by family members of gamblers, since the anger level is not related to a specific trait of the FMG but to a current situation that may be triggering anger. Longitudinal studies are necessary to verify whether, as the gambling problem subsides, changes also occur in the symptomatology of family members.

Secondly, the relationship between the study variables in the clinical population was analyzed, and significant relationships were found between them. These results are in line with previous studies where it has been indicated that an increase in dysfunctional emotion-regulation strategies such as rumination would produce a parallel increase in anxiety and depression symptoms (Schäfer et al., 2017; Thorsteinsson et al., 2019). Similarly, rumination has been reported to maintain and aggravate negative stressors and is strongly linked to major depressive disorder (de Rosa & Keegan, 2018; García et al., 2017) and anger (Balsamo, 2010). However, these results are novel in family members of pathological gamblers. Thus, the findings suggest that, as family members of pathological gamblers increase the use of emotion-regulation strategies based on rumination, psychological symptomatology such as depression, anxiety, and anger would increase in parallel. In the case of anger, psychological symptolotalogy would be associated above all with the phenomenological experience of anger and its expression, rather than with difficulties in its management. Likewise, in the present study, anger was related to anxiety and depression, which is consistent with previous studies showing that anger is associated with and favors the presence of such psychopathological problems (de Bles et al., 2019; Dowling, 2014).

Third, the mediating role of rumination between anger, depression, and anxiety was confirmed. These results are consistent with previous studies in other populations showing that the tendency to ruminate mediates the relationship between anger and depression. That is, they found that anger and depression were strongly linked, just as rumination was strongly associated with anger and depression (Balsamo, 2010). Another study mentioned that anger rumination played an important role in the relationship between anger and depression (Besharat et al., 2013). Likewise, Krause et al. (2018) found that rumination mediated the relationship between pathological gambling and depression in gamblers. In terms of anxiety, rumination has been found to mediate the relationship between anger and social anxiety (Trew & Alden, 2009). The variable that determined the mediating role of rumination was brooding, which consists of negativistic rumination or the tendency to brooding oneself and situations by comparing the current situation to some standard that has not been met (Thanoi & Klainin-Yobas, 2015). Brooding, feelings of guilt, and self-criticism are strongly related to depression (González et al., 2017; Tabardillo & Andrade, 2020). In the case of gambling, these results are little studied. In other addictions, however, family members of alcohol users have also been found to exhibit feelings of guilt and a tendency to brooding (Mendoza et al., 2016). Likewise, it was found that the type of kinship did not play a significant role, so these experiences could be generalizable to family members regardless of the type of relationship. The problem of pathological gambling affects the whole family system, partner, parents, siblings, etc. (Dowling, 2014). Perhaps because the relationship to the gambler, and the personal situation and personality of the family member are more important than the type of kinship.

This study has a number of limitations. Firstly, it is a cross-sectional study, which does not allow us to establish causal relationships between the study variables. This affects the validity of the mediational analyses. Future longitudinal studies could help to clarify the mechanisms that occur in the family members of pathological gamblers and the time frame in which they appear. In addition, the information was collected through self-reported questionnaires, so there may be a social desirability bias in participants' responses. On another hand, the sociodemographic characteristics of the sample could differ between the two groups, which could bias the results obtained in the mean difference analyses. In turn, one of the samples was composed of family members attending care sessions for family members of gamblers, who might differ in their characteristics from other family members. Finally, there is the possibility that the emotional problems shown by the family members were present before the onset of the gambling behavior and were not triggered by it. Moreover, they may have facilitated the development of pathological gambling in their family members.

In conclusion, these results show that family members of gamblers show higher levels of anger, anxiety, depression, and rumination than the general population, and that rumination mediates between anger and anxiety and depression. These results are of interest for clinical intervention in groups of family members, and highlight the importance of attending to and containing the emotional experiences they experience in the course of their family member's gambling problem.

There is a clear need for research that explores the impacts of pathological gambling on family members. This would allow for the expansion of knowledge and the identification of protective factors to develop and design specific and effective treatments for family members to reduce the negative effects of gambling. It would also allow the identification of effective strategies for managing problem gambling in the family, the development and evaluation of new therapeutic approaches, as well as the development of education-based problem gambling prevention programs, which are important ways to address risky behaviors among adolescents to prevent an escalation of problem behaviors in adulthood.

## Data Availability

The datasets generated during and/or analysed during the current study are available from the corresponding author on reasonable request.
